# The Atlantic Monthly

**Published:** 1866-05

**Authors:** 


The Atlantic Monthly, is placed regularly upon our table. It is devoted to Literature, Science, Art, and Politics. It is now in the ninth year of its existence, and has just completed its sixteenth volume. It numbers among its regular contributors nearly all the first names in American literature, and its pages present each month an agreeable variety of the best literature, prose and poetry. The publishers propose "to furnish to the readers of the *Atlantic* the best Essays, the best Stories, the best Poems, that can be procured; to make the magazine so rich and varied in matter, so pure and attractive in style, and so high and unexceptionable in moral tone, that no intelligent person can afford to do without it, and that it shall be regarded by all the thoughtful class of American readers as one of the *necessaries* of life."

